# Association Between the c.34C > T (rs17602729) Polymorphism of the AMPD1 Gene and the Status of Endurance and Power Athletes: A Systematic Review and Meta-Analysis

**DOI:** 10.1007/s40279-025-02202-9

**Published:** 2025-05-07

**Authors:** Jihan Kartibou, El Mokhtar El Ouali, Juan Del Coso, Anthony C. Hackney, Abderrazak Rfaki, Ayoub Saeidi, Rawad El Hage, Urs Granacher, Abdelhalem Mesfioui, Hassane Zouhal

**Affiliations:** 1 Department of Biology, Laboratory of Biology and Health, Ibn Tofail University of Kenitra, Kenitra, Morocco; 2 Sports Science Research Team, Institute of Sports Sciences, Hassan I University, Settat, Morocco; 3 Sport Sciences Research Centre, Rey Juan Carlos University, Fuenlabrada, Spain; 4 Department of Exercise and Sport Science, University of North Carolina, Chapel Hill, NC USA; 5 National Center for Scientific and Technical Research (CNRST), Rabat, Morocco; 6 Department of Physical Education, Faculty of Arts and Sciences, University of Balamand, PO Box 100, Tripoli, Lebanon; 7 Department of Physical Education and Sport Sciences, Faculty of Humanities and Social Sciences, University of Kurdistan, Sanandaj, Kurdistan Iran; 8 Department of Sport and Sport Science, Exercise and Human Movement Science, University of Freiburg, Freiburg, Germany; 9 Movement, Sport, Health and Sciences Laboratory (M2S). UFR-STAPS, University of Rennes 2-ENS Cachan, Av. Charles Tillon, 35044 Rennes Cedex, France; 10 Institut International Des Sciences du Sport (2IS), 35850 Irodouer, France

## Abstract

**Background:**

Previous research has shown that variants in the *AMPD1* gene, which encodes the adenosine monophosphate deaminase 1 (AMPD1) protein, may affect energy supply of the muscle and fatigue resistance during high-intensity exercise. A single nucleotide substitution in this gene, specifically a cytosine-to-thymine substitution (c.34C > T; rs17602729), results in a nonsense mutation that causes a deficiency in the AMPD1 protein. Deficiency of the AMPD1 protein due to this polymorphism can influence exercise performance, ultimately affecting the likelihood of reaching the status of elite endurance or power athlete.

**Objective:**

This systematic review and meta-analysis aimed to investigate the distribution of CC, CT, and TT genotypes of the *AMPD1* c.34C > T polymorphism (rs17602729) in endurance and power athletes to assess potential associations between this polymorphism and elite athlete status.

**Methods:**

Studies investigating genotype distribution in the *AMPD1* c.34C > T (rs17602729) polymorphism in endurance and/or power athletes were searched for in four electronic databases (PubMed, Web of Science, Scopus, Science Direct). The studies were selected and the genotypic and allelic frequencies of the *AMPD1* c.34C > T (rs17602729) polymorphism were extracted if data for endurance and/or power athletes were compared with controls (non-athletes). Meta-analyses were computed using fixed or random effects models to calculate odds ratios (OR) with confidence interval (95% CI). Heterogeneity of the meta-analyses was reported using *I*^2^ statistics.

**Results:**

After examining 1229 studies on the distribution of the *AMPD1* c.34C > T (rs17602729) polymorphism in endurance and/or power athletes, 20 studies were considered eligible to be included in our meta-analysis. The studies were conducted in 11 different countries, including 5717 participants. There was a higher frequency of the CC genotype (OR 1.72; 95% CI 1.40–2.12; *p* < 0.00001) in endurance athletes compared with non-athletic controls with a lower frequency of CT (OR 0.61; 95% CI 0.49–0.75; *p* < 0.00001) and TT genotypes in endurance athletes versus non-athletic controls (OR 0.43; 95% CI 0.19–0.97; *p* = 0.04). A higher frequency of the CC genotype was also observed in power athletes compared with controls (OR 2.17; 95% CI 1.69–2.78; *p* < 0.00001) with a lower frequency of the CT (OR 0.51; 95% CI 0.39–0.65; *p* < 0.00001) and TT genotypes (OR 0.25; 95% CI 0.09–0.68; *p* = 0.007) in power athletes compared with controls. Overall, the genotype distribution of the *AMPD1* c.34C > T polymorphism (rs17602729) was similar in endurance and power athletes (OR between 0.76 and 1.39; *p* = 0.47–0.72).

**Conclusion:**

Our findings indicate that the CC genotype was overrepresented in endurance and power athletes compared with controls, suggesting that possessing two copies of the C allele of the *AMPD1* c.34C > T (rs17602729) polymorphism may be associated with a 1.72–2.17 times greater likelihood of achieving elite or sub-elite athlete status in disciplines reliant on aerobic and anaerobic metabolic pathways. No statistically significant differences were found in the *AMPD1* genotype distribution between endurance and power athletes.

**Supplementary Information:**

The online version contains supplementary material available at 10.1007/s40279-025-02202-9.

## Key Points

**Table:** 

The muscle-specific isoform 1 of adenosine monophosphate deaminase (AMPD1) protein is a key regulator of the energy metabolism within muscle fibers. The AMPD1 protein is encoded by the *AMPD1* gene, located on the short arm of the first chromosome (1p13.1). The most researched variation in the *AMPD1* gene entails the change of cytosine to thymine at nucleotide 34 of the coding sequence located in exon 2 (c.34C > T, rs17602729).
Individuals carrying the mutant polypeptide sequence, whether homozygous (TT) or heterozygous (CT), exhibit lower and intermediate levels of myoadenylate deaminase compared with CC homozygotes.
This study revealed a significant association between the c.34C > T (rs17602729) polymorphism of the *AMPD1* gene and the status of being classified as an elite endurance or power athlete compared with non-athletic controls.
Findings revealed that the CC genotype was overrepresented in endurance athletes versus controls, suggesting that possessing two copies of the C allele of the *AMPD1* c.34C > T (rs17602729) polymorphism may be associated with a higher possibility of becoming an elite/sub-elite endurance athlete.
Similarly, the CC genotype was overrepresented in power athletes versus controls while CT and TT genotypes were underrepresented. This suggests that individuals with one or two copies of the T allele of the *AMPD1* c.34C > T (rs17602729) have a lower probability of becoming elite/sub-elite power athletes.

## Introduction

In recent years, the impact of genetics on athletic performance has become one of the most debated research topics in the fields of sports science and medicine. Several studies have highlighted the existing link between genetic variants and exceptional physical and physiological abilities, revealing associations between certain genetic polymorphisms and athletic performance [1–7]. To date, more than 200 genes and polymorphisms have been identified as having a significant impact on athletic performance and elite athlete status [8, 9]. Additionally, it has been suggested that the genetic influence on some traits could explain up to 90% of the variability of anaerobic performance, up to 60% of the variability of cardiorespiratory markers, and up to 70% of the variability in maximal muscular strength [10].

The muscle-specific isoform 1 of adenosine monophosphate deaminase (AMPD1) protein is a key regulator of the energy metabolism within muscle fibers, modifying the balance of myokine reactions in favor of adenosine triphosphate (ATP) production (2ADP → AMP + ATP) [11]. Additionally, the AMPD1 protein facilitates the deamination of AMP, converting it to inosine monophosphate (IMP), (AMP + H_2_O → IMP + NH_3_) [12], which thus plays an important role in regulating the energy available to skeletal muscle during exercise [13]. The AMPD1 protein has therefore been associated with the replenishment of ATP in the event of muscle fatigue, guaranteeing sustained muscle performance. Furthermore, the AMPD1 protein reaction is the starting point of the purine nucleotide cycle, playing a central role in the recovery of adenine nucleotides and the determination of energy needs [12, 14]. As such, the AMPD1 protein is involved in restoring adenosine molecules and modulating levels of IMP, AMP, adenosine diphosphate (ADP), and ATP in skeletal muscle during physical activity and exercise [15].

The AMPD1 protein is encoded by the *AMPD1* gene, located on the short arm of the first chromosome (1p13.1) [12]. This gene contains several single nucleotide polymorphisms (SNPs) that can alter protein characteristics and functionality. Generally, a SNP is a simple variation at a single position in the DNA sequence where one nucleotide is altered [16]. SNPs are the most common types of genetic variation in humans, occurring naturally throughout the genome, with millions identified to date [17]. These variations are present in both coding and non-coding regions of DNA and can potentially influence protein sequences. Importantly, these genetic variations can have a significant impact on physical and physiological attributes, including muscle composition, susceptibility to injury, and overall athletic performance, as well as the potential to achieve elite athlete status, especially if they are in genes that codify proteins with key functions for exercise physiology such as the AMPD1 [7, 18–21]. In the *AMPD1* gene, the most studied variation involves a cytosine-to-thymine substitution at nucleotide 34, known as the c.34C > T polymorphism (rs17602729) [22]. This substitution leads to the transformation of the amino acid glutamine into a stop codon [23]. This causes a premature interruption of protein synthesis, rendering the AMPD1 protein catalytically inactive [23]. Individuals carrying the mutant T allele, whether homozygous (TT) or heterozygous (CT), have reduced myoadenylate deaminase activity [14]. TT homozygotes have the lowest activity, while CT heterozygotes have intermediate levels [14]. Conversely, individuals carrying two copies of the normal (wild-type) C allele (the CC genotype) have normal AMPD1 protein activity in skeletal muscle [14, 24]. The minor allele frequency (MAF) for this polymorphism is low, as the T allele results in the production of a non-functional protein. However, it varies significantly, ranging from 0.1–0.5% in individuals of African or East Asian descent to 12.3% in individuals of European descent [25]. Studies on the c.34C > T (rs17602729) polymorphism in the *AMPD1* gene indicate that power and endurance-oriented athletes generally have a lower prevalence of the T allele than their non-athletic counterparts [26–29]. This means that the presence of the T allele in the *AMPD1* gene could decrease the likelihood of becoming an elite endurance or power athlete [30, 31]. Possession of the T allele can be associated with another phenotype such as reduced peak oxygen uptake (VO_2_ peak) and less affinity to endurance training [32], which may be limiting factors for endurance performance [33]. Lastly, the absence of the AMPD1 protein has been linked to an increased frequency of mild forms of post-exercise myopathy, characterized by increased fatigue and a high prevalence of muscle cramps [33]. Although the study regarding the influence of the *AMPD1* 34T allele on limiting exercise performance has been a topic of interest in research, no previous study has summarized the findings of previous studies on this polymorphism. Here, we primarily aimed to review and meta-analyze studies on the distribution of genotypes of the *AMPD1* c.34C > T (rs17602729) polymorphism in endurance and power athletes compared with controls (e.g., non-athletes), and to explore the potential associations between this polymorphism and elite athlete status. As a second goal, we examined potential associations between the c.34C > T (rs17602729) polymorphism of the *AMPD1* gene and the status of being classified as an endurance or power athlete. Based on the relevant literature [30, 31], we hypothesized that the proportion of individuals with the CT and TT genotypes would be lower in endurance and power athletes than in non-athletic controls.

## Methods

Our study was previously registered on PROSPERO with the registration number CRD42023420853. The meta-analysis was conducted in strict accordance with the guidelines outlined in the Cochrane Handbook for Systematic Reviews and Meta-Analysis of Interventions [34]. Furthermore, our literature search adhered to the guidelines of the PRISMA (Preferred Reporting Items for Systematic Reviews and Meta-Analyses) statement [35].

### Definitions of Terms

Endurance sports (aerobic-based sports), typically involve physical activities that last longer than 5 min and are characterized by exercise intensities near or below VO_2_ peak. These types of sport rely heavily on the aerobic energy system, in which the respiratory and circulatory systems play a critical role in the provision of energy through oxidative metabolism. Sustained effort in these activities requires a constant supply of ATP, the primary energy currency of the cell, to support muscle contractility and overall performance [1, 36]. Anaerobic sports typically include short-duration power disciplines with single or repeated bursts of high-intensity actions. These activities demand strong and powerful muscular contractions, primarily supported by the anaerobic energy system. These anerobic pathways encompass glycolysis and the ATP/phosphocreatine system, providing rapid and brief energy release mechanisms. Through these metabolic pathways, rapid bursts of muscle activations are achieved, essential for activities such as sprinting, jumping, weightlifting, and other high-intensity efforts [37].

### Eligibility Criteria

Only studies investigating the association between the c.34C > T (rs17602729) polymorphism of the *AMPD1* gene and athlete status (endurance and power-oriented) were included in our analysis. Accordingly, we selected studies examining the genotypic and allelic distribution of the c.34C > T (rs17602729) polymorphism in athletes from anaerobic and/or aerobic-based sport disciplines who were compared with non-athletic controls. Therefore, in conducting this meta-analysis, we identified studies that met the following criteria: (1) publication in peer-reviewed journals, (2) elite and sub-elite athletes participating in endurance or power-based sports, (3) inclusion of individuals aged 14 years or older, (4) incorporation of healthy non-athletic individuals serving as controls, and (5) provision of data regarding the frequency of the *AMPD1* c.34C > T (rs17602729) polymorphism, distinguishing CC, CT, and TT genotypes separately. We excluded studies that (1) did not meet the minimal criteria for an experimental study design, such as case reports, (2) did not satisfy the minimum criteria for sample classification as elite or sub-elite athletes, (3) were not written in English, or (4) only presented data on the frequency of the *AMPD1* polymorphism as CC genotype versus T allele carriers or as TT genotype versus C allele carriers. Additionally, we excluded systematic or narrative review articles and studies with an elevated risk of bias concerning intervention or outcome.

### Literature Search Strategy

Literature searches were conducted in four electronic databases: PubMed, Web of Science, Scopus, Science Direct, from their inception to April 2024, with no year restriction applied to the search syntax. Search terms, including key terms and synonyms, searched in the databases, were combined using the operators ‘AND’, ‘OR’ and ‘NOT’ as follows: CONCEPT 1 (‘adenosine monophosphate deaminase’ OR ‘*AMPD1*’) OR (‘*AMPD1* gene’ OR ‘*AMPD1* c.34C > T (rs17602729) polymorphism’ OR ‘adenosine monophosphate deaminase c.34C > T (rs17602729)’ OR ‘*AMPD1* genotype’) AND CONCEPT 2 (‘endurance elite athletes*’ OR ‘endurance sub-elite athletes*’ OR ‘endurance athletes*’ OR ‘endurance individual sports athletes*’ OR ‘endurance team sports athletes*’) OR CONCEPT 3 (‘power elite athletes*’ OR ‘power sub-elite athletes*’ OR ‘power athletes*’ OR ‘power individual sports athletes*’ OR ‘power team sports athletes*’). The search for published studies was conducted independently by two authors (JK and EMEO) and any discrepancies were resolved by discussion with all authors.

### Study Selection

Two researchers, JK and EMEO, reviewed and selected the studies according to the a priori defined inclusion and exclusion criteria. Articles appearing potentially relevant based on their titles were further screened by consulting their abstract. If an article was considered eligible on the basis of its abstract, the full text was then reviewed for further evaluation. In the event of disagreement between the two investigators regarding the inclusion of an article, a consensus meeting was organized with all the authors.

### Data Extraction

Following the application of inclusion and exclusion criteria, we conducted data extraction from the studies to gather information in accordance with the PRISMA methodology, focusing on a modified version of the PECOS elements (participants, exposure, comparator, outcomes, and study design) [38], Table 1. Key data extracted from each study encompassed study particulars (author, publication year, and country), characteristics of the study population (sample size, age, and gender of participants), the method used to determine the genotype of the *AMPD1* c.34C > T (rs17602729) polymorphism, along with the count/frequency of participants exhibiting CC, CT, and TT genotypes in each type of population under investigation. Where this information was not available, frequencies were calculated using raw data where possible. Data extraction was conducted by JK and EMEO.

**Table 1:** PECOS (participants, exposure, comparator, outcomes, study design) approach

PECOS element	Details
Participants	Elite and sub-elite athletes, non-athletes and healthy subjects
Exposure	*AMPD1* C34T polymorphism genotyping
Comparators	Athletes (endurance and power sports athletes) versus controls (non-athletes)
Outcomes	Genotypic and allelic frequencies of *AMPD1* C34T polymorphism
Study design	Cross sections, case-controls, cohorts and all interventional studies

### Quality Assessment

We used the Newcastle–Ottawa Scale (NOS), which is recognized as a reliable and valid tool, to assess the methodological quality of observational studies (including cohort and case–control studies) [39]. The NOS can be used to classify studies according to their methodological quality based on three main criteria including (i) participant selection, (ii) comparability of groups, and (iii) exposure/outcomes which is essential to obtain reliable and robust conclusions. The NOS assigns an overall score based on the three domains mentioned above. Each domain is subdivided into items, and each item can be given a binary score of 0 or 1 point. A score of 1 indicates that the study meets the assessment criteria, while a score of 0 indicates that the study does not meet the assessment criteria. Thus, the highest score for a study rated with the NOS is 9 points. However, any study that obtained a score higher than 6 on the NOS was considered to be of high quality. Two reviewers (EMEO and JK) independently performed the quality assessment. In the absence of consensus on the quality of a study, all authors were consulted to make a final assessment decision.

### Statistical Analyses

Data from each study (endurance and power athletes and controls) were used to determine the overall effect of the *AMPD1* c.34C > T (rs17602729) polymorphism across all study populations using a meta-analytic approach. In particular, the number of athletes with CC, CT, and TT genotypes in each study was recorded and the OR with 95% CI was calculated for each comparison. The threshold for statistical significance was set at a value of *p* < 0.05. The *I*^2^ statistical test was used to assess the degree of heterogeneity of results between studies within the same comparison. In this regard, *I*^2^ values of 25%, 50%, and 80% were considered to represent a low, moderate, and elevated level of heterogeneity, respectively. The level of heterogeneity was used to select an appropriate model for each analysis. For *I*^2^ values of < 50%, we used a fixed effects model, and for *I*^2^ values of > 50%, we used a random effects model. For sensitivity analysis, we used both fixed- and random-effects models, performing the analysis independently of study heterogeneity. Additionally, we conducted a leave-one-out analysis by systematically excluding one study at a time to assess the impact of the omitted data on the OR and heterogeneity. In accordance with Cochrane guidelines [40], we identified any publication bias in our meta-analysis by visual inspection of the funnel plot of each comparison. This meta-analysis was performed using the free online software Cochrane Review Manager (RevMan) 5.4.1.

## Results

### Selection of Studies and Characteristics of Included Studies

**Fig. 1:**
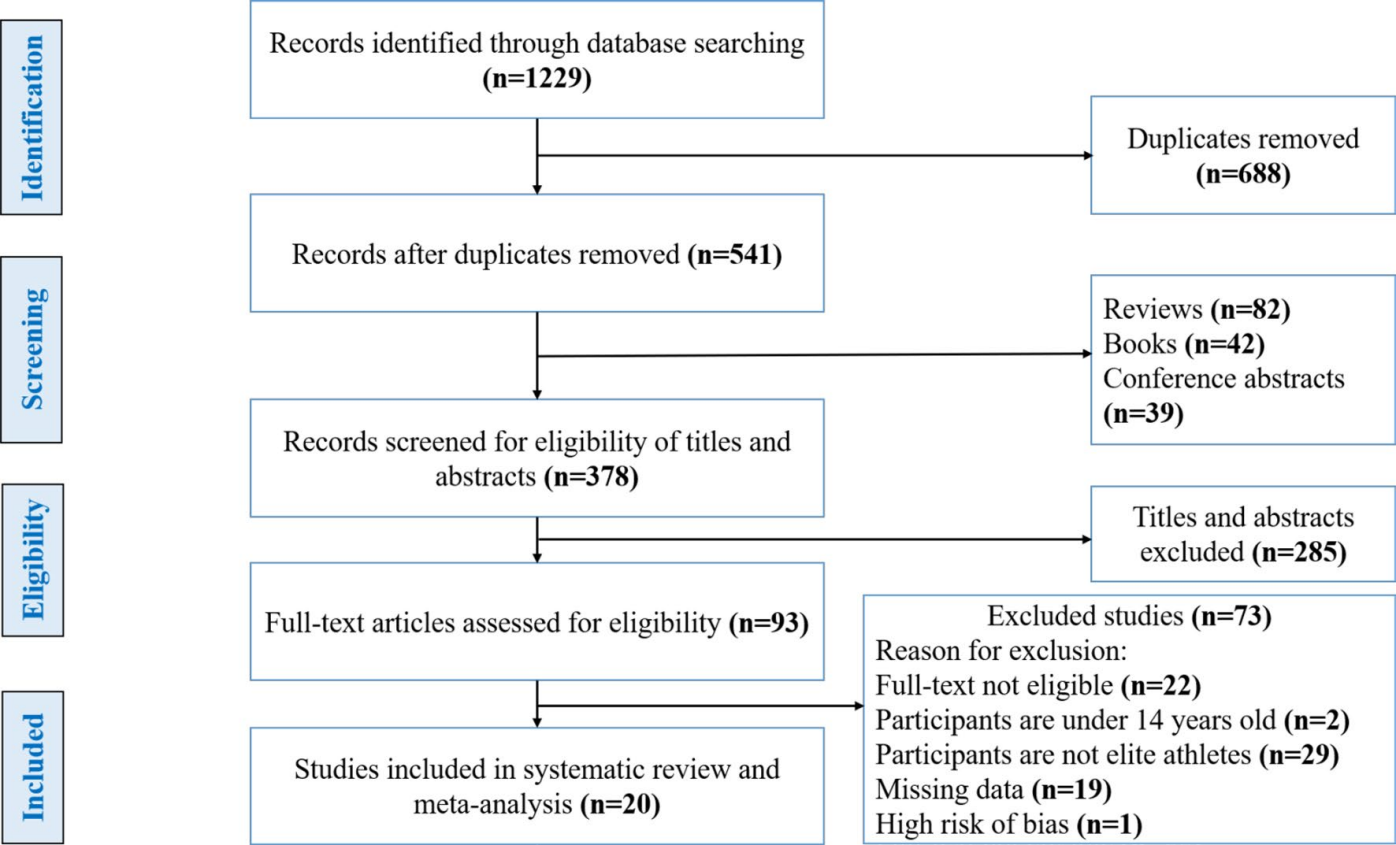
Flowchart for screening eligible studies for systematic review and meta-analysis

Our initial database search (PubMed, Scopus, Science Direct and Web of Science) yielded a total of 1229 publications, of which 688 were duplicates; 82 reviews, 39 conference abstracts, 42 books, and 285 articles were deemed ineligible on the basis of title and abstract. Subsequently, 93 articles were reviewed in detail and 73 were excluded based on the results of the full-text assessment for the following reasons: full-text not eligible (*n* = 22), participants were under age 14 years (*n* = 2), participants were not elite or sub-elite athletes (*n* = 29), missing data (*n* = 19), high risk of bias (*n* = 1). Based on inclusion and exclusion criteria and quality assessment, 20 eligible studies were included in our systematic review and meta-analysis (Fig. 1). These studies were conducted in 11 different countries: Poland, Spain, Brazil, Bulgaria, Russia, Lithuania, Australia, Turkey, Israel, the Czech Republic, and Greece, with a sample of 5717 participants (3078 athletes and 2639 controls). Of the 20 included studies, 14 studies examined only males, and 6 studied both females and males in their sample. The general characteristics of the 20 studies included in the systematic review and meta-analysis are summarized in Table 2.

**Table 2:** Characteristics of the 20 included studies in the systematic review and meta-analysis

Study	Newcastle–Ottawa Scale	Country	Sex	Sample size	Age, years (mean ± SD or range)	Participants	Genotyping
Cieszczyk et al. 2011 [30]	8	Poland	Men	Elite athletes (*N* = 44)	23–61	Elite rowers, non-elite rowers and controls (students from the University of Szczecin)	PCR–RFLP
				Non-elite athletes (*N* = 83)	18–41		
				Controls (*N* = 251)	19–23		
Cieszczyk et al. 2012 [29]	8	Poland	Men	Elite athletes (*N* = 158)	19–34	Power-oriented athletes, sub-elite and controls (students from the University of Szczecin)	PCR–RFLP
				Sub-elite athletes (*N* = 134)	19–23		
				Controls (*N* = 160)			
Dionísio et al. 2017 [46]	6	Brazil	Men	Athletes (*N* = 220)	14–20	Professional minor league of the soccer team	qPCR
Djarova et al. 2013 [43]	7	Bulgaria	Men	Athletes (*N* = 5)	50 ± 14.6	Elite high-altitude mountaineers and controls (students)	PCR–RFLP
				Controls (*N* = 72)	21.5 ± 1.8		
Fedotovskaya et al. 2013 [12]	8	Russia	Men and women	Athletes (*N* = 305)		High-speed and strength sports and controls (high school students)	PCR–RFLP
				Men: 252	21.2 ± 4.3		
				Women: 53	25.7 ± 3.1		
				Controls (*N* = 499)			
				Men: 139	19.6 ± 2.1		
				Women: 360	18.0 ± 3.3		
Ginevičienė et al. 2014 [11]	8	Lithuania	Men and women	Athletes (*N* = 204)	22.0 ± 6.3	Endurance and power-oriented athletes and controls (healthy unrelated individuals)	PCR–RFLP
				Men: 160			
				Women: 44			
				Controls (*N* = 260)	36.2 ± 7.2		
Grealy et al. 2015 [13]	6	Australia	Men	Athletes (*N* = 196)	25 ± 5.4 21.1 ± 2	Elite endurance triathletes	PCR–RFLP
Gronek et al. 2018 [31]	6	Poland	Men	Athletes (*N* = 180)	18–65	Experienced half marathon runners	PCR–RFLP
Horozoglu et al. 2021 [47]	6	Turkey	Men	Athletes (*N* = 33)	18–25	Super amateur league football players	qPCR
Maestro et al. 2022 [48]	6	Spain	Men	Athletes (*N* = 122)	Data not shown	Professional soccer players	PCR–RFLP
Meckel et al. 2012 [42]	8	Israel	Men and women	Athletes (*N* = 155) Men: 119 Women: 36 Controls (*N* = 142) Men: 100 Women: 42	35.9 ± 12.2	Endurance and sprint athletes and non-athletic healthy individuals	PCR–RFLP
Muniesa et al. 2010 [49]	8	Spain	Men	Athletes (*N* = 141) Rowers: 39 Olympic-class runners: 52 Professional road cyclists: 50 Controls (*N* = 132)	Data not shown	Runners, cyclists, rowers and healthy, non-athletic individuals	PCR–RFLP
Petr et al. 2022 [50]	8	Czech Republic	Men	Athletes (*N* = 99)	25.4 ± 4.51	Elite soccer players and controls (healthy subjects)	PCR–RFLP
				Controls (*N* = 107)	18–65		
Pranckeviciene et al. 2021 [51]	8	Lithuania	Men and women	Athletes (*N* = 180) Men: 130 Women: 50 Controls (*N* = 255)	26.4 ± 6.7	Elite athletes	PCR–RFLP
Ruiz et al. 2009 [15]	8	Spain	Men	Athletes (*N* = 46) Endurance runners: 14 Professional road cyclists: 32 Controls (*N* = 123)	Data not shown	Endurance runners, professional road cyclists and controls (healthy male non-athletic individuals)	PCR–RFLP
Santiago et al. 2010 [44]	8	Spain	Men	Athletes (*N* = 53) World-class rowers: 39 National-class rowers:15 Controls (*N* = 123)	Data not shown	World-class rowers, national-class rowers and controls (healthy male non-athletic individuals)	PCR–RFLP
Tsianos et al. 2009 [52]	6	Greece	Men and women	Athletes (*N* = 438) Men: 417 Women:21	38.4 ± 8.3	Olympus marathon runners	PCR–RFLP
Varillas Delgado et al. 2020 [41]	8	Spain	Men	Athletes (*N* = 123)		Professional road cyclists, elite endurance runners and controls (non-athlete participants)	PCR–RFLP
				Professional road cyclists: 75	25.8 ± 4.2 (18–42)		
				Elite endurance runners: 48	27.9 ± 5.1 (19–42)		
				Controls (*N* = 122)			
Varillas Delgado et al. 2022 [53]	8	Spain	Men	Endurance athletes (*N* = 160) Professional football players (*N* = 132) Non-athletes (*N* = 160)	Data not shown	Professional cyclists, elite long-distance runners, professional football players and non-athlete individuals (non-smokers, and not suffering from chronic or acute diseases, or obesity)	PCR–RFLP
Varillas Delgado et al. 2022 [54]	6	Spain	Men and women	Athletes (*N* = 100) Men: 50 Women: 50	Data not shown	Elite endurance athletes	PCR–RFLP

*PCR–RFLP* polymerase chain reaction-restriction fragment length polymorphism, *qPCR* quantitative polymerase chain reaction

### Study Quality Assessment

Following the assessment using the Newcastle–Ottawa Scale (NOS), 7 studies achieved a score of 6 points, 1 study scored 7 points, and 12 studies obtained 8 points (Table 3). Overall, the quality of the studies included in our systematic review and meta-analysis was deemed satisfactory, with a mean score of 7.25 ± 0.94.

**Table 3:** Quality assessment of included studies using the Newcastle–Ottawa Scale [39]

Newcastle–Ottawa Scale quality assessment for cohort studies										
Study	Selection				Comparability		Outcome			Quality score
	1	2	3	4	5	6	7	8	9	
Cieszczyk et al. 2011 [30]	1	1	1	1	1	1	1	0	1	8
Cieszczyk et al. 2012 [29]	1	1	1	1	1	1	1	0	1	8
Dionísio et al. 2017 [46]	1	0	1	1	1	0	1	0	1	6
Djarova et al. 2013 [43]	0	1	1	1	1	1	1	0	1	7
Fedotovskaya et al. 2013 [12]	1	1	1	1	1	1	1	0	1	8
Ginevičienė et al. 2014 [11]	1	1	1	1	1	1	1	0	1	8
Grealy et al. 2015 [13]	1	0	1	1	1	0	1	0	1	6
Gronek et al. 2018 [31]	1	0	1	1	1	0	1	0	1	6
Horozoglu et al. 2021 [47]	1	0	1	1	1	0	1	0	1	6
Maestro et al. 2022 [48]	1	0	1	1	1	0	1	0	1	6
Meckel et al. 2012 [42]	1	1	1	1	1	1	1	0	1	8
Muniesa et al. 2010 [49]	1	1	1	1	1	1	1	0	1	8
Petr et al. 2022 [50]	1	1	1	1	1	1	1	0	1	8
Pranckeviciene et al. 2021 [51]	1	1	1	1	1	1	1	0	1	8
Ruiz et al. 2009 [15]	1	1	1	1	1	1	1	0	1	8
Santiago et al. 2010 [44]	1	1	1	1	1	1	1	0	1	8
Tsianos et al. 2009 [52]	1	0	1	1	1	0	1	0	1	6
Varillas Delgado et al. 2020 [41]	1	1	1	1	1	1	1	0	1	8
Varillas Delgado et al. 2022 [53]	1	1	1	1	1	1	1	0	1	8
Varillas Delgado et al. 2022 [54]	1	0	1	1	1	0	1	0	1	6

1: Representativeness of the exposed cohort, 2: Selection of the nonexposed cohort, 3: Ascertainment of exposure, 4: Demonstration that outcome of interest was not present at start of study, 5 and 6: Comparability of cohorts on the basis of the design or analysis, 7: Assessment of outcome, 8: Was follow-up long enough for outcomes to occur and 9: Adequacy of follow up of cohorts

1: Was assigned when the study met the requested criteria

0: Was assigned when the study did not meet the requested criteria

### Meta-Regression and Cumulative Meta-Analysis

In this meta-analysis, the 20 eligible studies examined the frequency of different genotypes of the *AMPD1* c.34C > T (rs17602729) polymorphism in endurance athletes of various disciplines (such as rowers, cyclists, high-altitude mountaineers, triathletes, half-marathon runners, and marathon runners) as well as in power athletes of various disciplines (including sprinters, soccer players and powerlifting athletes), comparing these frequencies with those of non-athletic controls.

**Fig. 2:**
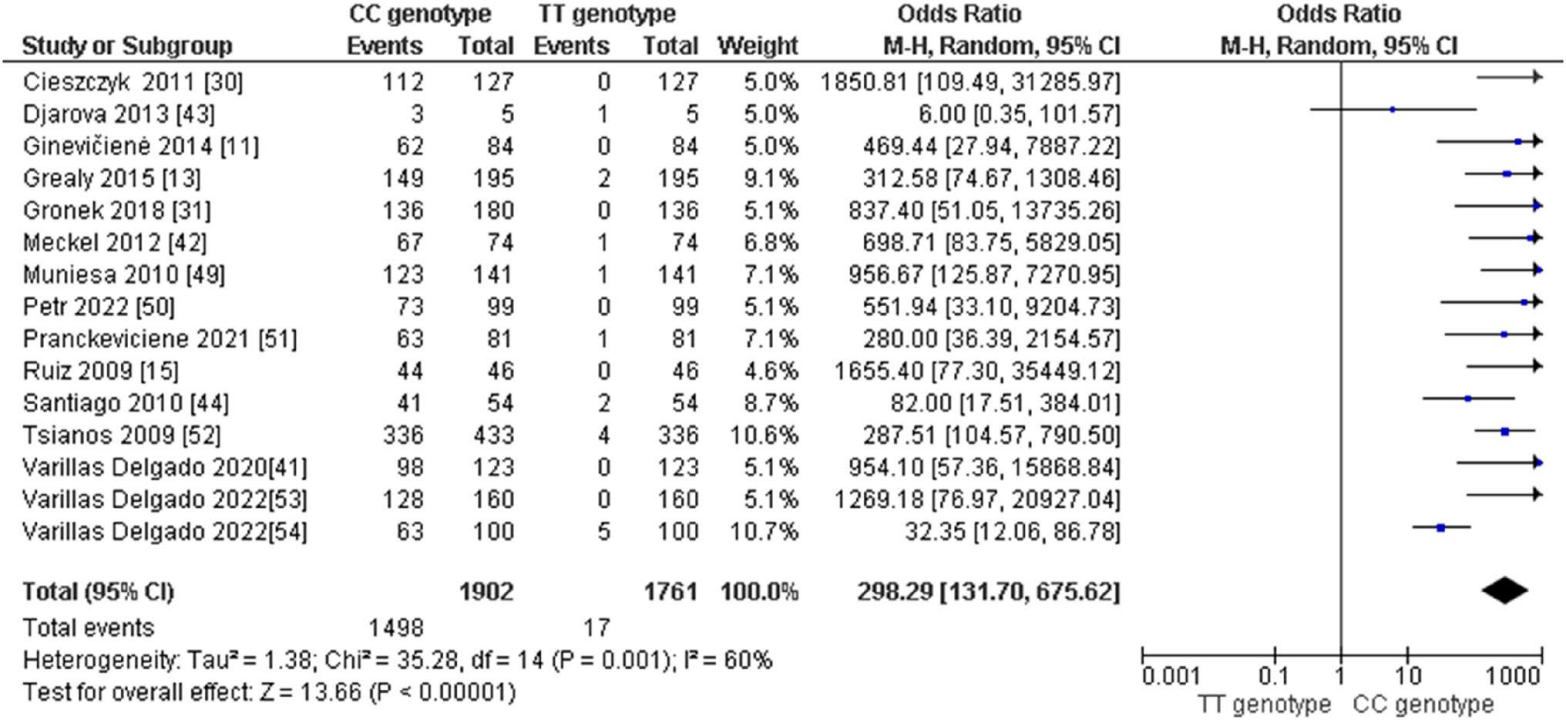
Forest plot of the comparison between frequencies of CC and TT genotypes in endurance athletes

**Fig. 3:**
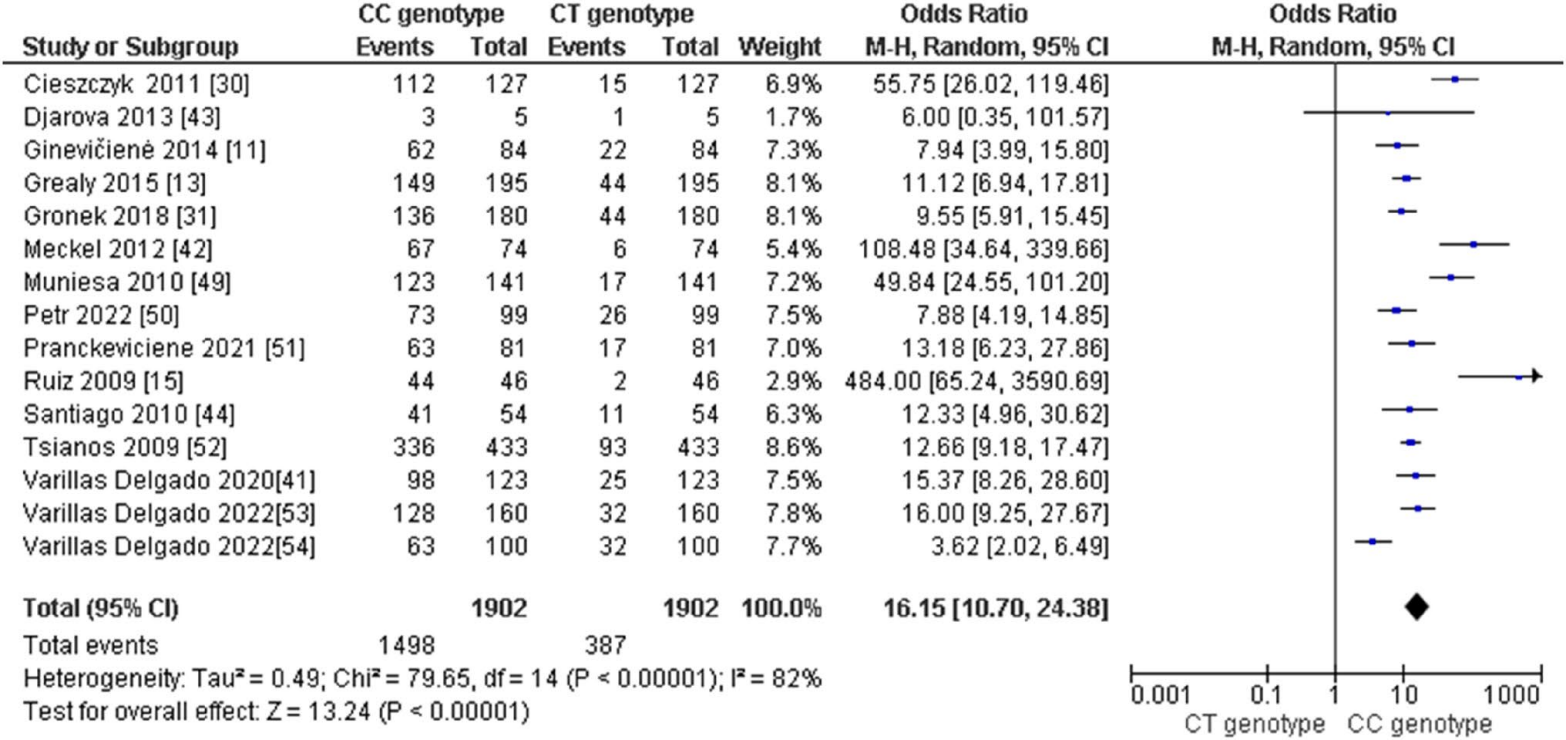
Forest plot of the comparison between frequencies of CC and CT genotypes in endurance athletes

**Fig. 4:**
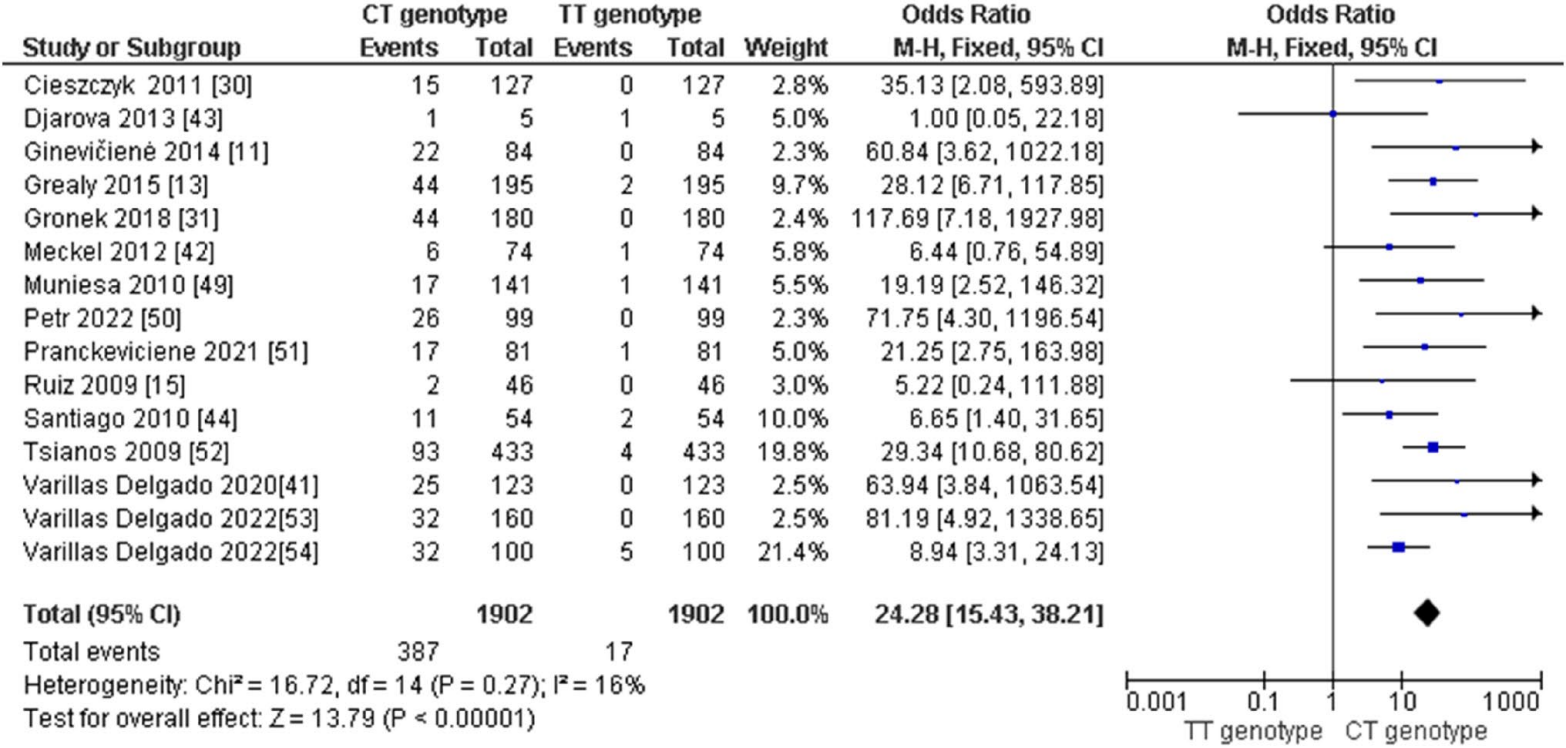
Forest plot of the comparison between frequencies of CT and TT genotypes in endurance athletes

**Fig. 5:**
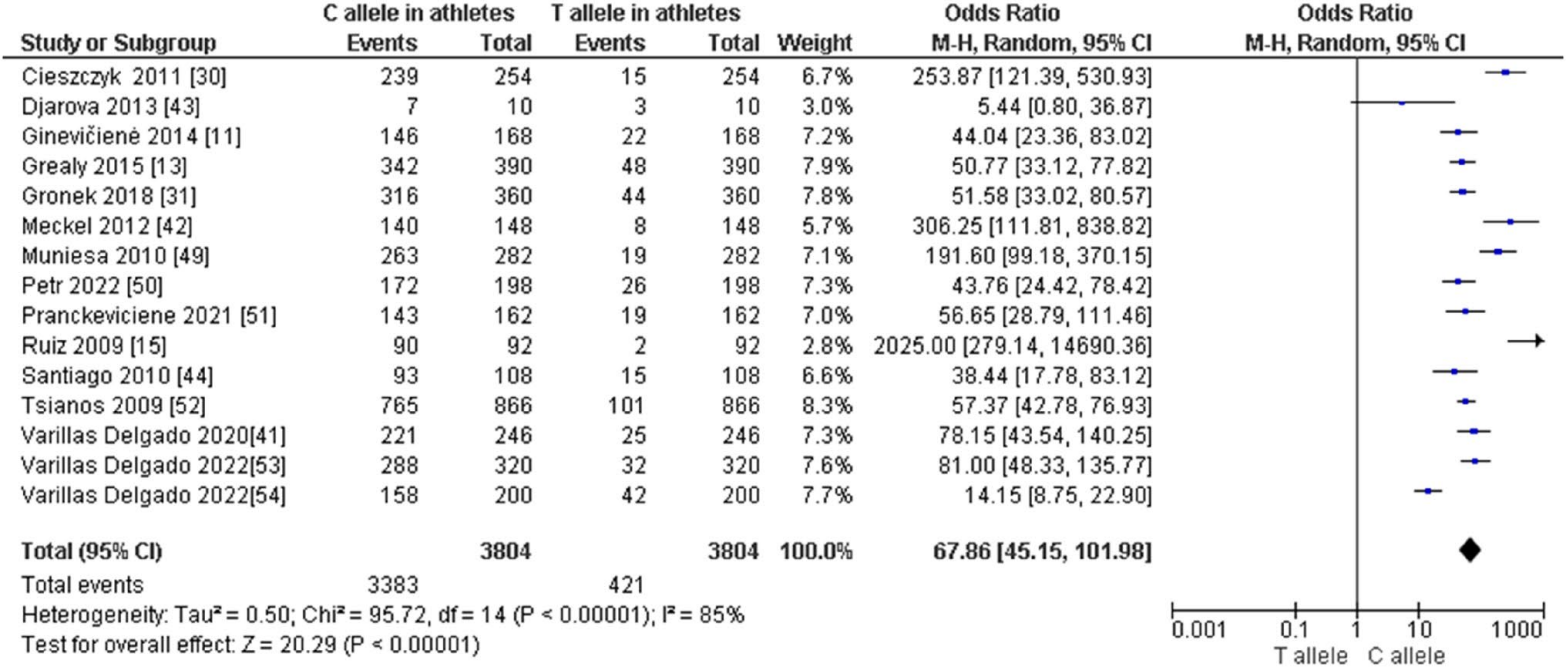
Forest plot of the comparison of the C allele versus the T allele in endurance athletes

Regarding the distribution of *AMPD1* CC, CT, and TT genotypes in endurance athletes, we obtained the following results: (i) the pooled data showed that the frequency of the CC genotype was significantly higher compared with the TT genotype in endurance athletes (odds ratio [OR] 298.29; 95% confidence interval [CI] 131.70–675.62; *p* < 0.00001 and 60% heterogeneity, Fig. 2), (ii) the frequency of the CC genotype was significantly higher than that of the CT genotype (OR 16.15; 95% CI 10.70–24.38; *p* < 0.00001 and 82% heterogeneity, Fig. 3) and (iii) the frequency of the CT genotype was higher than that of the TT genotype (OR 24.28; 95% CI 15.43–38.21; *p* < 0.00001 and 16% heterogeneity, Fig. 4). Regarding the allelic frequency, we also found a dominance of the C allele compared with the T allele in endurance athletes (OR 67.86; 95% CI 45.15–101.98; *p* < 0.00001 and 85% heterogeneity, Fig. 5).

**Fig. 6:**
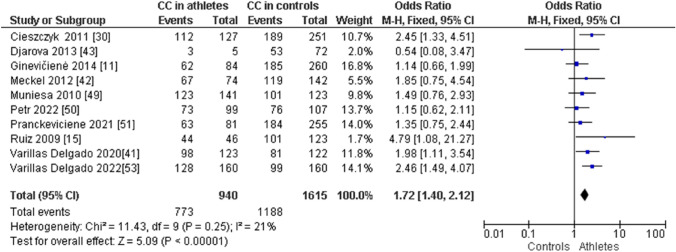
Forest plot of the comparison of CC genotype frequency in endurance athletes versus controls

**Fig. 7:**
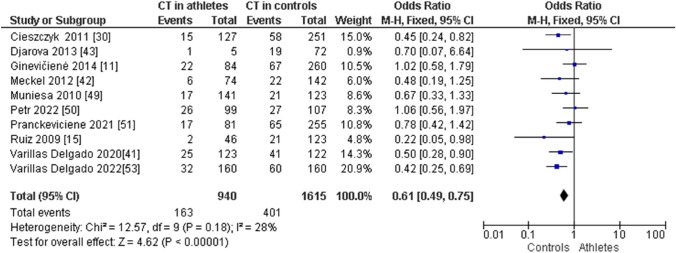
Forest plot of the comparison of CT genotype frequency in endurance athletes versus controls

**Fig. 8:**
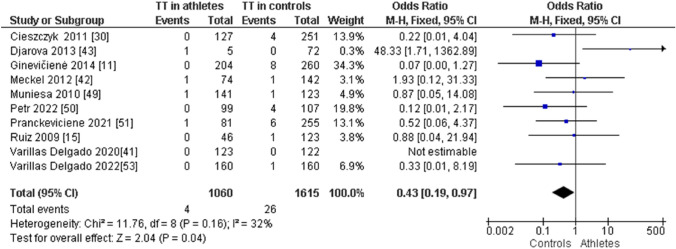
Forest plot of the comparison of TT genotype frequency in endurance athletes versus controls

**Fig. 9:**
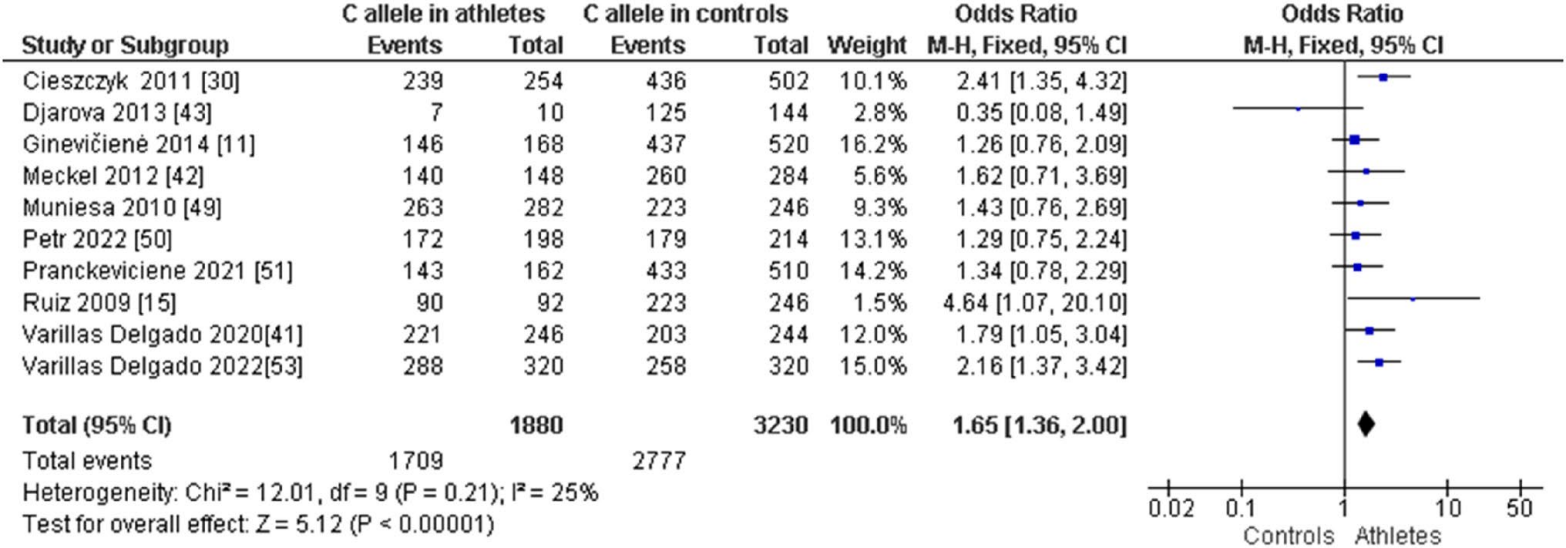
Forest plot of the comparison of the C allele in endurance athletes versus controls

**Fig. 10:**
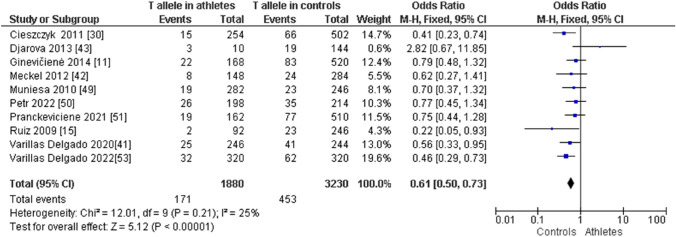
Forest plot of the comparison of the T allele in endurance athletes versus controls

When comparing endurance athletes with non-athletic controls, we observed a higher frequency of CC in endurance athletes: CC (OR 1.72; 95% CI 1.40–2.12; *p* < 0.00001, and 21% heterogeneity, Fig. 6). However, the frequency of CT (OR 0.61; 95% CI 0.49–0.75; *p* < 0.00001 and 28% heterogeneity, Fig. 7) and TT (OR 0.43; 95% CI 0.19–0.97; *p* = 0.04 and 32% heterogeneity, Fig. 8) was lower in endurance athletes than in non-athletic controls. There was a dominance of the C allele in endurance athletes compared with non-athletic controls (OR 1.65; 95% CI 1.36–2.00; *p* < 0.00001 and 25% heterogeneity, Fig. 9) and a lower frequency of the T allele in endurance athletes compared with non-athletic controls (OR 0.61; 95% CI 0.50–0.73; *p* < 0.00001 and 25% heterogeneity, Fig. 10).

**Fig. 11:**
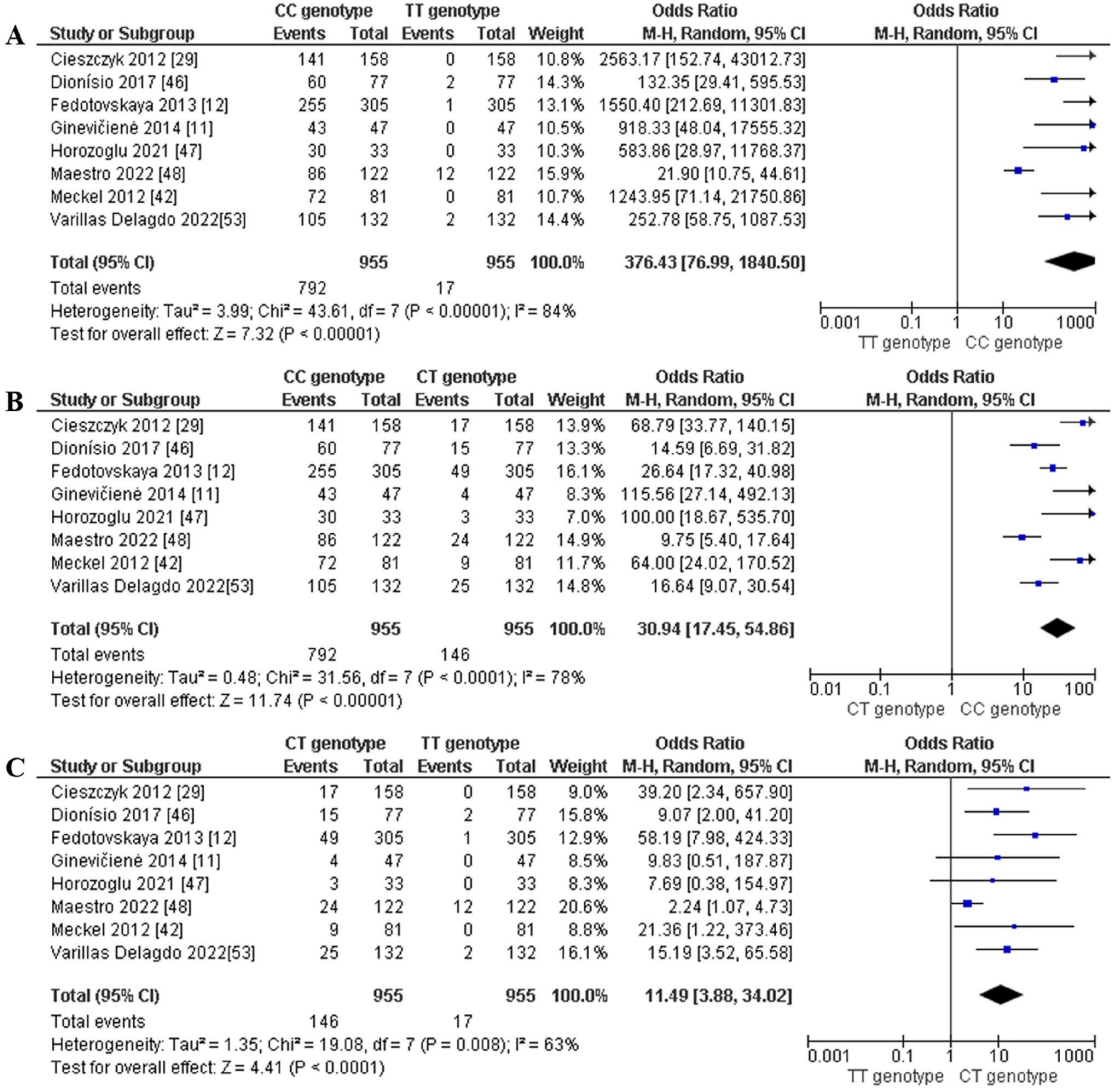
Forest plot of genotype frequency comparison in power athletes. **A** (CC vs TT), **B** (CC vs CT) and **C** (CT vs TT)

**Fig. 12:**
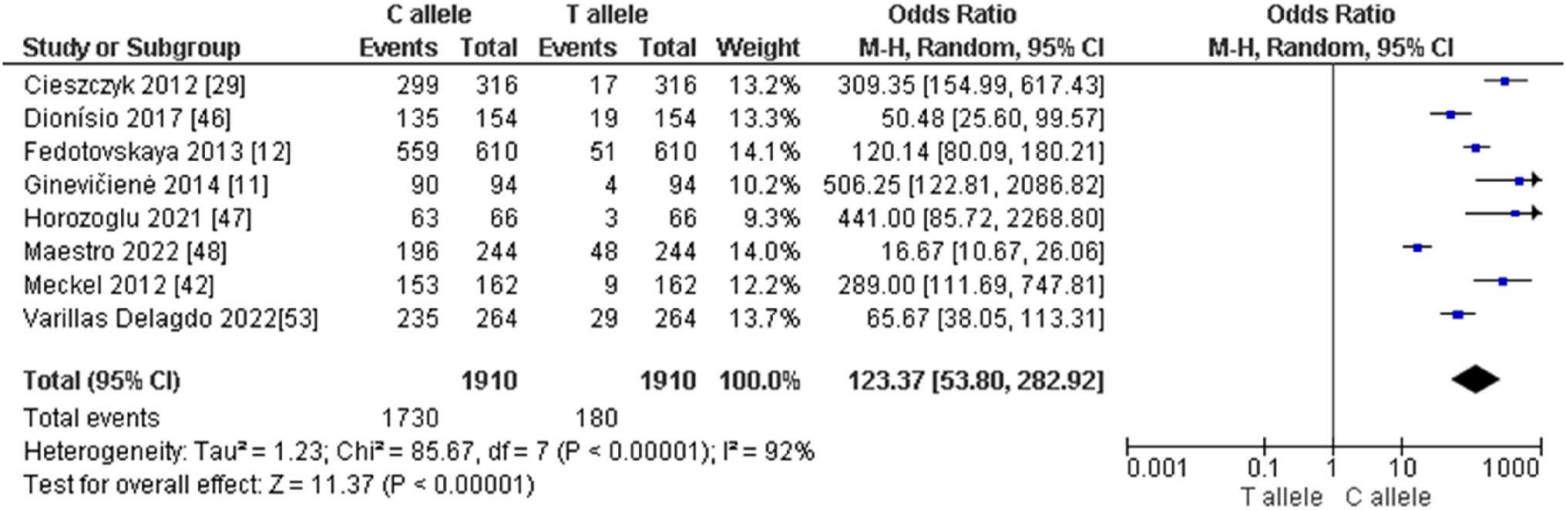
Forest plot of the comparison of the C allele versus the T allele in power athletes

For power athletes, our findings revealed the following outcomes on the distribution of the *AMPD1* c.34C > T (rs17602729) polymorphism: (i) the pooled data showed a significantly higher frequency of the CC genotype compared with the TT genotype in power athletes (OR 376.43; 95% CI 76.99–1840.50; *p* < 0.00001 and 84% heterogeneity, Fig. 11A), (ii) the frequency of the CC genotype was significantly higher than that of genotype CT (OR 30.94; 95% CI 17.45–54.86; *p* < 0.00001 and 78% heterogeneity, Fig. 11B) in power athletes, and (iii) the frequency of the CT genotype was higher when compared with the TT genotype in power athletes (OR 11.49; 95% CI 3.88–34.02; *p* < 0.0001 and 63% heterogeneity, Fig. 11C). We also found a dominance of the C allele compared with the T allele in power athletes (OR 123.37; 95% CI 53.80–282.92; *p* < 0.00001 and 92% heterogeneity, Fig. 12).

**Fig. 13:**
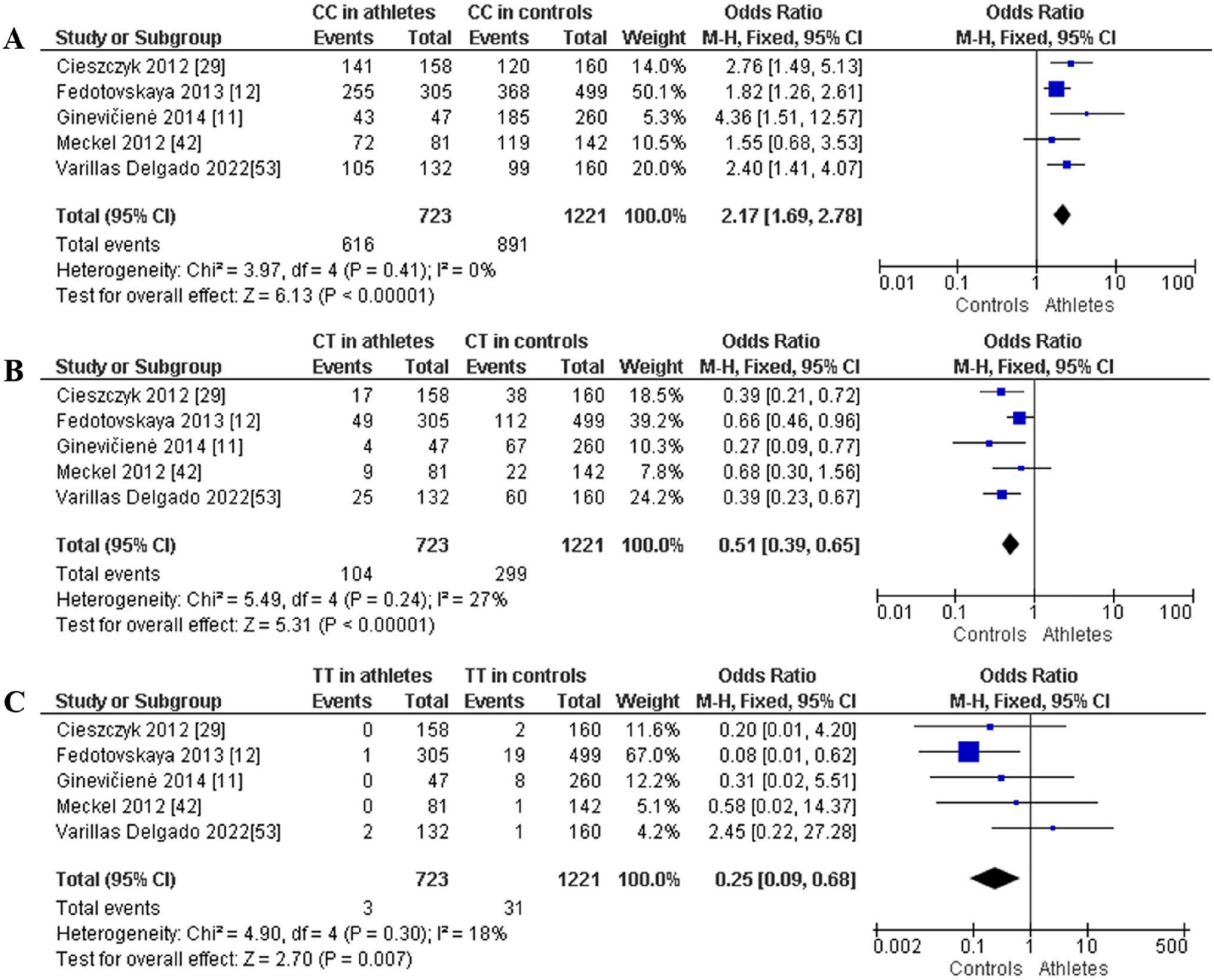
Forest plot of genotypic frequency comparison in power athletes versus controls. **A** CC in athletes vs controls, **B** CT in athletes vs controls, and **C **TT in athletes vs controls

**Fig. 14:**
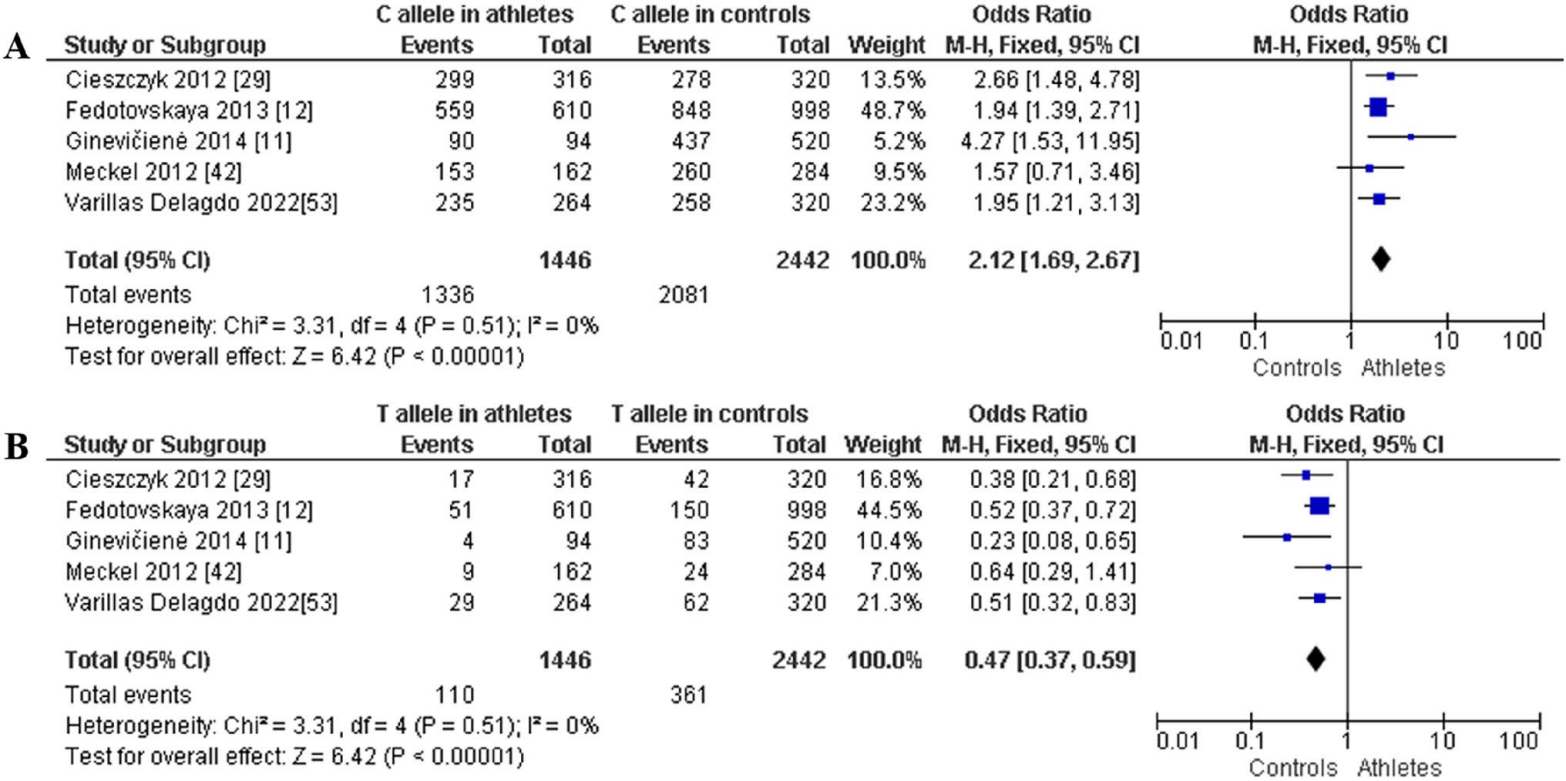
Forest plot of allelic frequency comparison between power athletes versus controls. **A** C allele in athletes vs controls and **B** T allele in athletes vs controls

Additionally, when comparing power athletes with non-athletic controls, we noted a higher frequency of CC in power athletes: CC (OR 2.17; 95% CI 1.69–2.78; *p* < 0.00001 and 0% heterogeneity, Fig. 13A) and lower frequency of CT (OR 0.51; 95% CI 0.39–0.65; *p* < 0.00001 and 27% heterogeneity, Fig. 13B) and TT genotypes (OR 0.25; 95% CI 0.09–0.68; *p* = 0.007 and 18% heterogeneity, Fig. 13C). Moreover, a dominance of the C allele was found in power athletes versus controls (OR 2.12; 95% CI 1.69–2.67; *p* < 0.00001, with 0% heterogeneity, Fig. 14A). In contrast, a lower frequency of the T allele was observed in power athletes compared with controls (OR 0.47; 95% CI 0.37–0.59; *p* < 0.00001 with 0% heterogeneity, Fig. 14B).

**Fig. 15:**
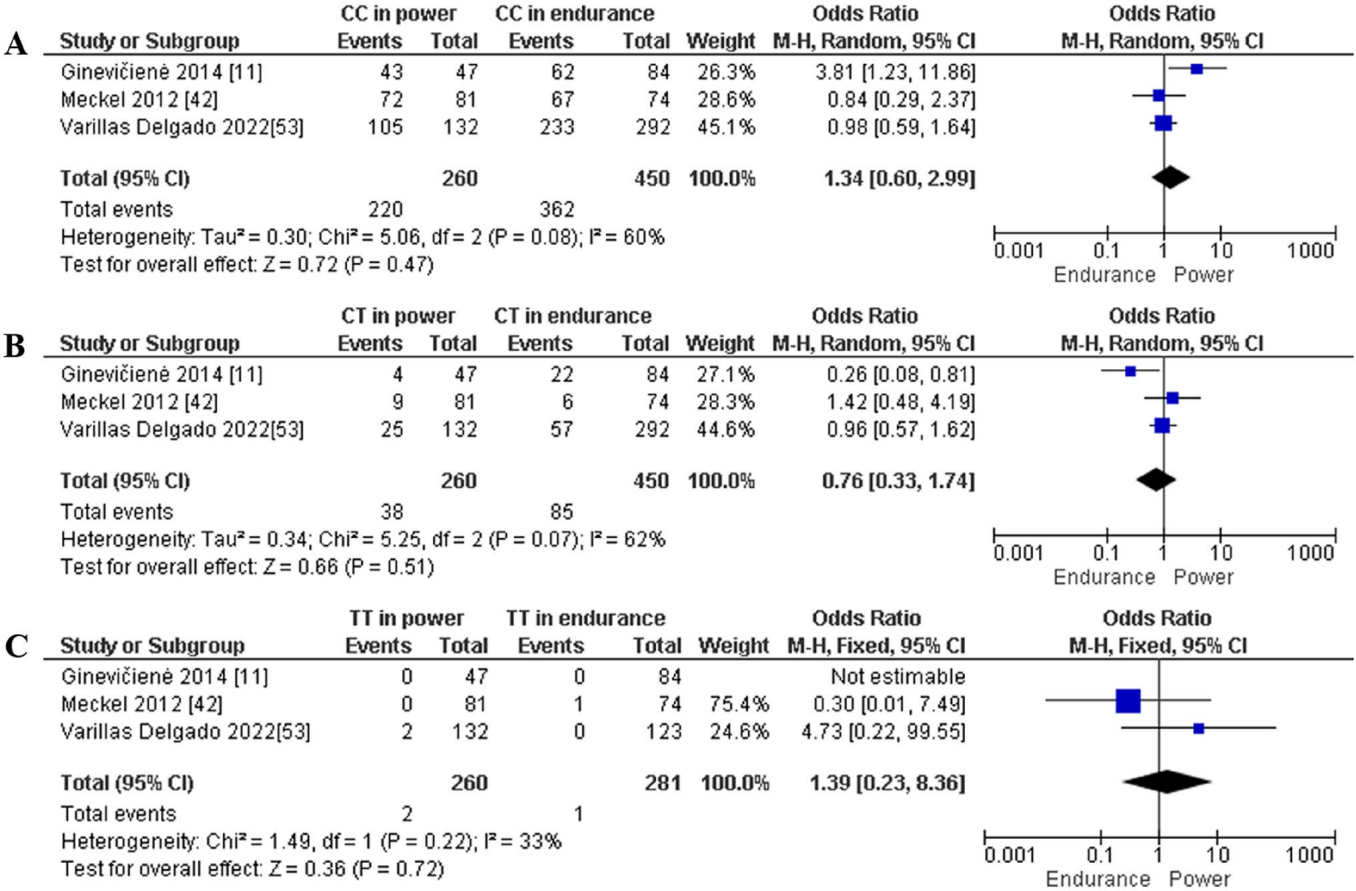
Forest plot of genotypic frequency comparison in power athletes versus endurance athletes. **A**: CC in power athletes vs endurance athletes, **B**: CT in power athletes vs endurance athletes and **C:** TT in power athletes vs endurance athletes

**Fig. 16:**
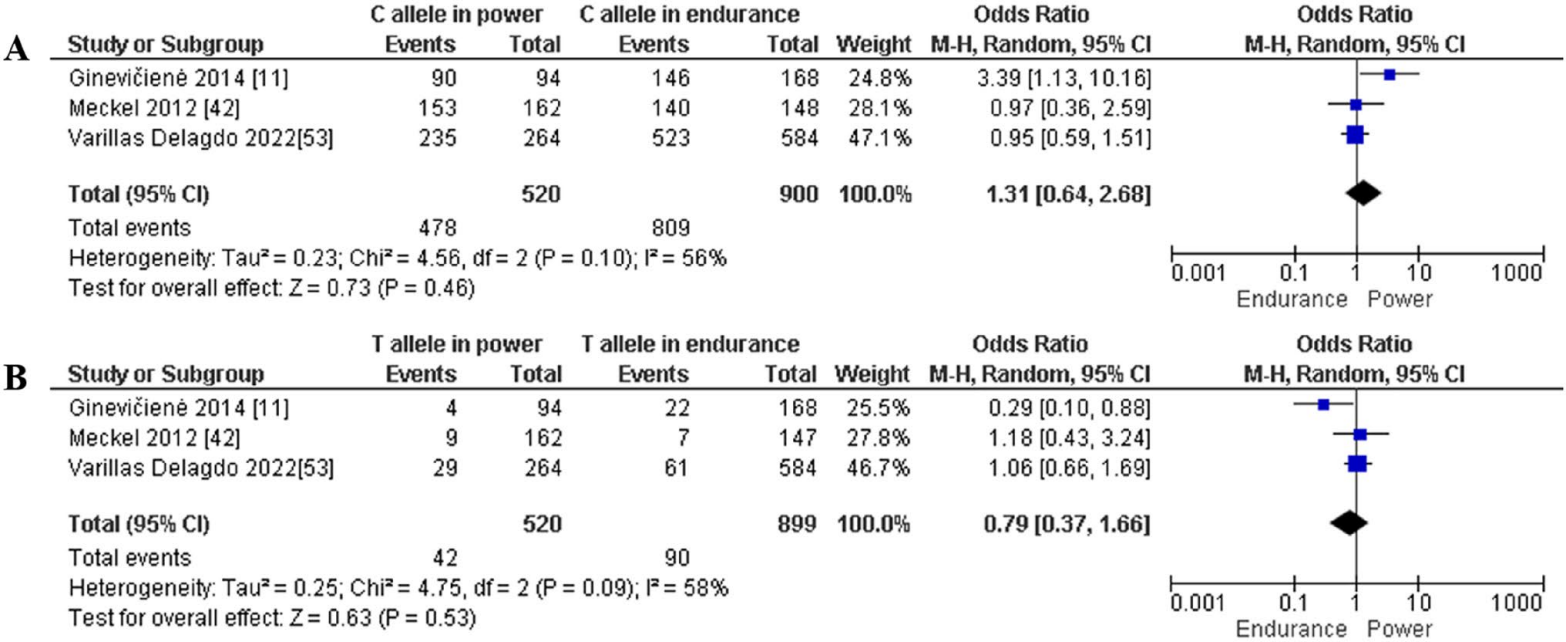
Forest plot of allelic frequency comparison between power athletes versus endurance athletes. **A**: C allele in power athletes vs endurance athletes and **B**: T allele in power athletes vs endurance athletes

Finally, the contrast between endurance and power athletes is based on three studies only. Our meta-analysis revealed a similar frequency of the CC (OR 1.34; 95% CI 0.60–2.99; *p* = 0.47 and 60% heterogeneity, Fig. 15A), CT (OR 0.76; 95% CI 0.33–1.74; *p* = 0.51 and 62% heterogeneity, Fig. 15B), and TT (OR 1.39; 95% CI 0.23–8.36; *p* = 0.72 and 33% heterogeneity, Fig. 15C) genotypes in endurance versus power athletes. Moreover, in terms of allele frequency, the difference between endurance and power athletes was not statistically significant for the C (OR 1.31; 95% CI 0.64–2.68; *p* = 0.46 and 56% heterogeneity, Fig. 16A) and T (OR 0.79; 95% CI 0.37–1.66; *p* = 0.53 and 58% heterogeneity, Fig. 16B) alleles.

### Sensitivity Analysis

The use of fixed-effects and random-effects models revealed consistent differences between genotypes and power/endurance versus non-athletic controls in all comparisons. The ORs, *p*-values, and heterogeneity measures for all pairwise comparisons remained largely unchanged when individual studies were excluded, which may suggest the robustness of our results. The studies included in this meta-analysis exhibit a general similarity and adhere to nearly identical experimental designs. Consequently, the relatively high study heterogeneity observed, particularly in certain comparisons, was mainly due to variations in sample size between studies.

### Publication Bias

To assess publication bias in our study, we used visual inspection of the funnel plot from which we concluded there was no publication bias in the analysis included in this review. All funnel plots for genotype frequency comparisons are provided in the Supplementary Appendix (see electronic supplementary material [ESM]).

## Discussion

This study aimed to review and meta-analyze studies on the distribution of genotypes of the *AMPD1* c.34C > T (rs17602729) polymorphism in endurance and power athletes versus non-athletic controls and to explore the potential associations between this polymorphism and elite/sub-elite athlete status. This study is novel as it is the first to aggregate findings from original research in the form of a meta-analysis on the genotype distribution of the *AMPD1* c.34C > T (rs17602729) polymorphism in athletes of different sporting disciplines in comparison with non-athletes. Furthermore, the meta-analysis offers insights into the higher probability of becoming an elite/sub-elite athlete in a specific domain for those with the CC genotype, compared with those with the CT and TT genotypes, independently of the type of sport discipline. The main results of our meta-analysis demonstrated a higher frequency of the CC genotype in endurance and power-oriented athletes compared with the CT and TT genotypes, confirming our initial hypothesis. In addition, a significant dominance of the C allele over the T allele was observed in all athletes (endurance and power athletes) versus non-athletic controls. Interestingly, the distribution of the *AMPD1* gene in endurance and power-oriented athletes followed the same hierarchical pattern (CC > CT > TT) with no differences between the types of sport disciplines. Collectively, this evidence suggests a positive association between the CC genotype and an increased probability of achieving elite/sub-elite athlete status in both endurance- and power-related sport disciplines. Conversely, individuals with one or two copies of the T allele of the *AMPD1* c.34C > T (rs17602729) polymorphism have a lower probability of becoming elite/sub-elite endurance and power athletes.

In endurance athletes, the frequency of the CC genotype was higher than in controls. In addition, the CT and TT genotypes were more frequent in controls than in endurance athletes. In terms of alleles, the frequency of the T allele was significantly lower in endurance athletes than in non-athletes, and inversely for the C allele, which was higher in endurance athletes than in controls. Cieszczyk et al. [30] found that the frequency of the T allele was significantly lower in Polish elite rowers than in controls and concluded that the T allele may negatively impact athletic performance. Additionally, the study of a group of professional cyclists and elite endurance runners revealed a higher frequency of the CC genotype compared with the control group [41]. Still, the genotype (CC, CT, and TT) and allele (C and T) frequencies were similar in the endurance athletes (marathon), sprint athletes (100 m), and non-athletic controls in groups of Israeli individuals, suggesting that the *AMPD1* c.34C > T (rs17602729) polymorphism was not associated with the status of the examined athletes, at least in this sample [42]. The authors of this study explained the lack of significance by the small effective sample size. Additionally, there was no significant difference between professional endurance athletes and the control group [31]. Interestingly, Djarova et al. [43] found a significantly higher frequency of the T allele (*p* = 0.003) in elite mountaineers (30%) compared with controls (13%), while the C allele was higher in non-athletes (87%) compared with elite athletes (70%). However, these results can be explained by the small number of elite athletes (5 mountaineers). Taken together, our findings confirm those from previous studies, suggesting that possessing the C allele of the *AMPD1* c.34C > T (rs17602729) polymorphism, particularly two copies of this allele, may be associated with a higher possibility of becoming an elite/sub-elite endurance athlete. The novelty of the current study lies in the fact that, for the first time, the positive association between the *AMPD1* CC genotype and endurance performance has been established through a comprehensive meta-analysis of the available literature on this topic. Possessing a single copy of the C allele (i.e., the CT genotype) was not enough to increase the likelihood of becoming an elite/sub-elite endurance athlete, likely because this genotype represents a lower activity of AMPD1 protein in comparison with CC individuals [14].

Concerning power athletes, our main findings showed a significantly lower frequency of the TT genotype and the T allele in the power athletes compared with controls. Consistent with our findings, a significant difference in the distribution of the *AMPD1* gene was found previously in Polish power athletes and non-athletic controls [29]. However, these results indicate a dominance of the C allele and a reduced frequency of the T allele in Polish elite power athletes [29]. Interestingly, this finding suggests that the C allele of the c.34C > T (rs17602729) polymorphism may contribute to achieving elite status in power-oriented sports [29]. Additionally, a significantly lower frequency of the T allele in power athletes than in non-athletic controls was demonstrated [7, 8]. Therefore, these results might suggest an association between the c.34C > T (rs17602729) polymorphism of the *AMPD1* gene and anaerobic muscle activity in athletes. The presence of the *AMPD1* C34 allele may serve as an indicator associated with affinity to speed and power-related sports [12]. Conversely, the findings of Meckel et al. [42] suggest that the *AMPD1* c.34C > T (rs17602729) polymorphism is not associated with elite athlete status, at least within the sample of Israeli athletes, as mentioned before. The results of this meta-analysis suggest a possible association between the presence of the *AMPD1* 34T allele and a lower probability of achieving elite power athlete status. However, this interpretation warrants caution due to the limited number of athletes analyzed, which restricts the generalizability of the findings. The reported constraints emphasize the importance of conducting additional studies. Future investigations should aim to explore the relationship between the *AMPD1* 34T allele and specific physical and physiological performance parameters in athletes from power-related sports, with the aim of validating and elucidating this potential link more comprehensively.

Overall, the literature presents conflicting results. Several studies suggest that individuals with at least one copy of the T allele may have a genetic tendency to compromise ATP production. This predisposition can lead to exercise intolerance, characterized by symptoms such as early fatigue during physical activity and delayed recovery of muscle strength and power [26, 44]. Therefore, the presence of the T allele in the *AMPD1* gene can be considered a limiting factor for achieving elite/sub-elite endurance or power athlete status. Interestingly, the practical application of this meta-analysis, including the consideration of the *AMPD1* 34T allele as a performance-limiting factor by athletes and coaches, remains uncertain. Sports performance and elite athlete status are complex traits influenced by multiple factors, and a single SNP, such as the *AMPD1* 34T allele, is unlikely to have a significant impact on its own. Talent in sport is the result of the interaction of genetic, environmental, and training-related factors. It is important to note that other genetic biomarkers, as well as physical, physiological, and environmental factors, also play an important role in determining an athlete’s status. Finally, while the study of individual SNPs provides valuable information, athletic success requires a holistic approach that integrates genetics assessment with other key determinants, such as training, nutrition, and psychological resilience. Collectively, measuring multiple genetic variants in a high number of performance-enhancing polymorphisms is essential to understand an athlete's potential genetic predisposition for exceptional performance in a specific discipline. However, this genetic predisposition should be considered alongside other factors, such as injury history, previous experience, and training performance outcomes, as part of the overall preparation strategy of athletes. Genetic assessment should never be used as a standalone tool for talent identification [45].

### Limitations

Our meta-analysis explores the potential association between the *AMPD1* c.34C > T (rs17602729) polymorphism and elite athlete status, with a particular focus on endurance and power disciplines. However, due to the limited number of studies on the *AMPD1* gene, we did not take ethnic and sex differences between athletes into account, which is a recognized limitation of our study. Additionally, due to the relatively small number of studies included in the meta-analysis, we categorized sports into two broad disciplines: endurance or power-related sports. This represents a limitation, as the sports within each category (endurance or power), while sharing similar characteristics, also have notable differences. For example, in the endurance athlete group, disciplines such as road cycling, rowing, and running were included. Although these sports share a common reliance on aerobic metabolism as the primary energy source during exercise, their unique demands may result in differing associations between the *AMPD1* gene and elite/sub-elite athlete status. Similarly, in the power athlete group, sprinters and team sport athletes were grouped together despite the distinct characteristics of these sports. In the future and as more studies become available on this topic, subgroup analyses should be conducted within the broader categories of endurance (e.g., runners vs cyclists) and power (e.g., sprinters vs team sport athletes) to better understand the specific genetic associations within each discipline. Another limitation of this systematic review with meta-analysis is that it focused solely on a single polymorphism within one gene. It is widely recognized that elite athletic performance depends on the presence of multiple advantageous variants across several candidate genes. Therefore, the association of the c.34C > T (rs17602729) polymorphism in the *AMPD1* gene with athletic performance is likely influenced by interactions with other genetic factors and it should be interpreted cautiously when data for only one polymorphism are available.

## Conclusions

The main results of this meta-analysis showed a significant predominance of the CC compared with the CT and TT genotypes in athletes from endurance and power-related sports. Furthermore, our analysis revealed a significant predominance of the CC genotype in endurance and power athletes compared with non-athletic controls. Possessing two copies of the C allele of the *AMPD1* c.34C > T (rs17602729) polymorphism was associated with a 1.72–2.17 times greater likelihood of achieving elite or sub-elite athlete status in disciplines reliant on aerobic (endurance) and anaerobic (power) metabolic pathways. The results therefore indicate a relationship between the c.34C > T (rs17602729) polymorphism of the *AMPD1* gene and the status of becoming an elite/sub-elite endurance and power athlete.

## Supplementary Information

Below is the link to the electronic supplementary material.

Supplementary file1 (DOCX 127 KB)

## Declarations

### Data Availability Statement

All data supporting the findings of this study are available in this published article.
