# Supplementary material for: Association Between the c.34C > T (rs17602729) Polymorphism of the AMPD1 Gene and the Status of Endurance and Power Athletes: A Systematic Review and Meta-Analysis
Source: Sports Med. 2025 May 7;55(6):1429–48. doi: 10.1007/s40279-025-02202-9 (PMC12152022; doi:10.1007/s40279-025-02202-9)
Supplement: Supplementary file 1 — Supplementary file1 (DOCX 127 KB) [file 40279_2025_2202_MOESM1_ESM.docx]

Supplementary Material

**Funnel plots**


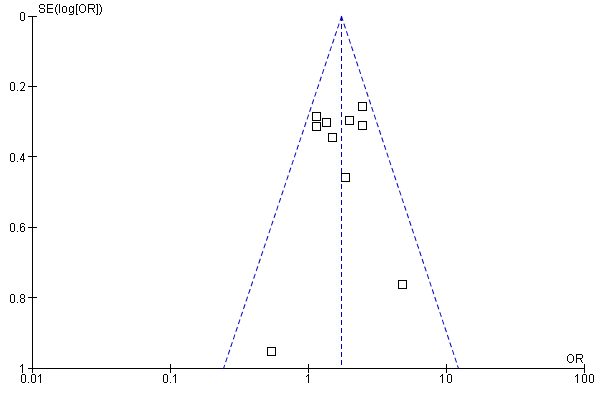


**Figure S1:** Funnel plot of the CC genotype expression in endurance athletes versus controls.

**
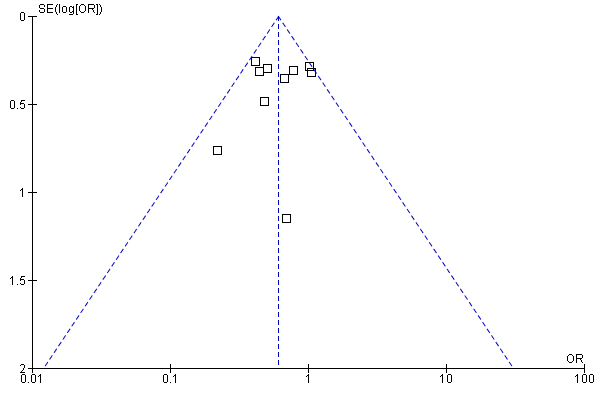
**

**Figure S2:** Funnel plot of the CT genotype expression in endurance athletes versus controls.


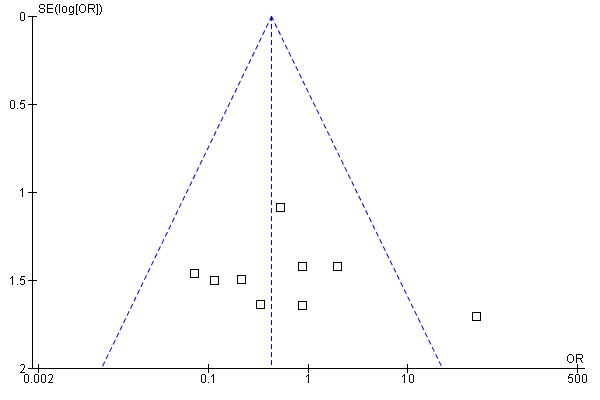


**Figure S3:** Funnel plot of the TT genotype expression in endurance athletes versus controls.


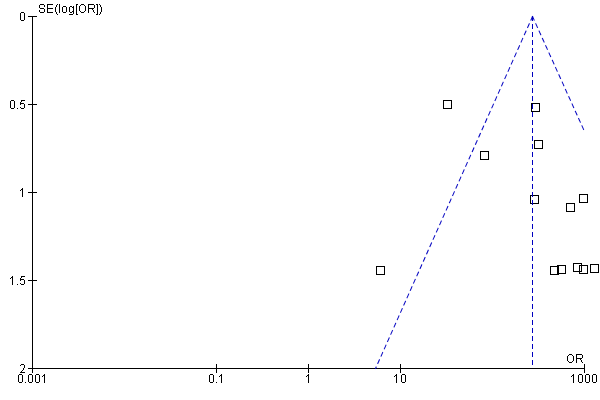


**Figure S4:** Funnel plot of the comparison between CC and TT genotypes in elite endurance athletes.


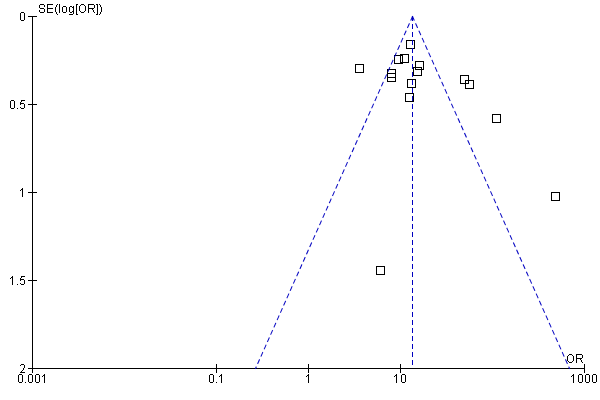


**Figure S5:** Funnel plot of the comparison between CC and CT genotypes in elite endurance athletes.


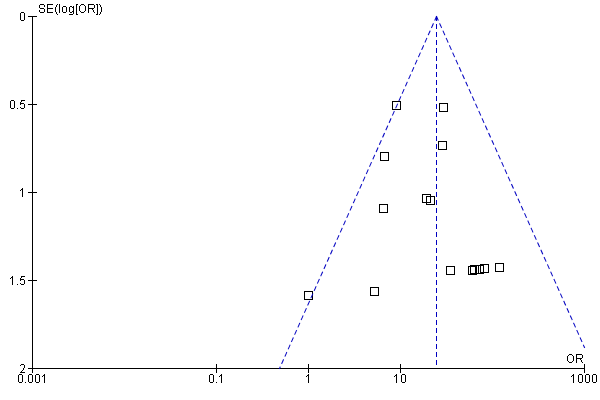


**Figure S6:** Funnel plot of the comparison between CT and TT genotypes in elite endurance athletes.


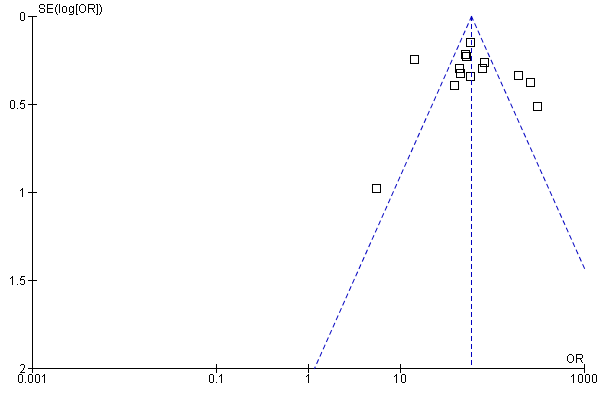


**Figure S7:** Funnel plot of the C allele versus T allele in elite endurance athletes.


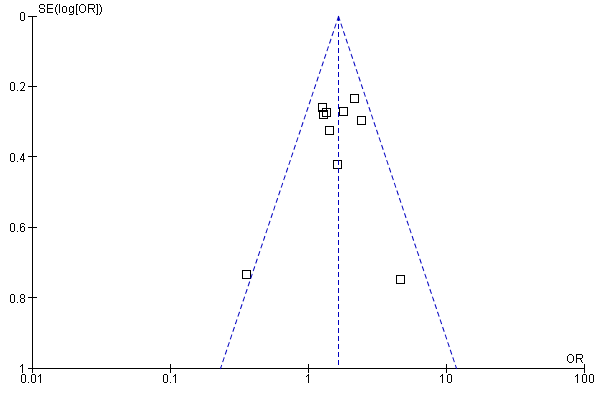


**Figure S8:** Funnel plot of the C allele in endurance athletes versus controls.


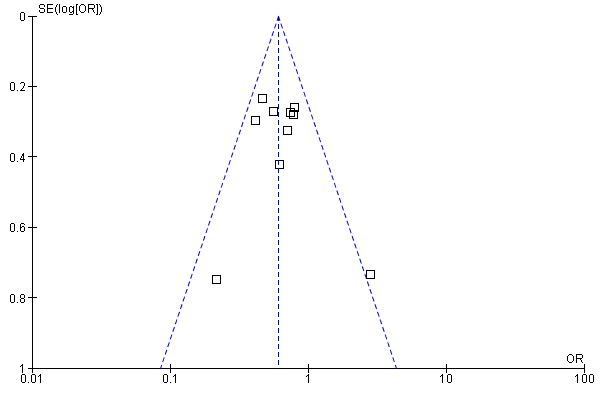


**Figure S9:** Funnel plot of the T allele in endurance athletes versus controls.


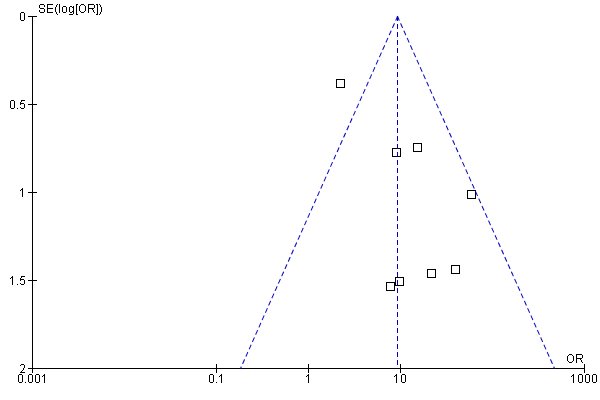


**Figure S10:** Funnel plot of the comparison between CC and TT genotypes in elite power athletes.


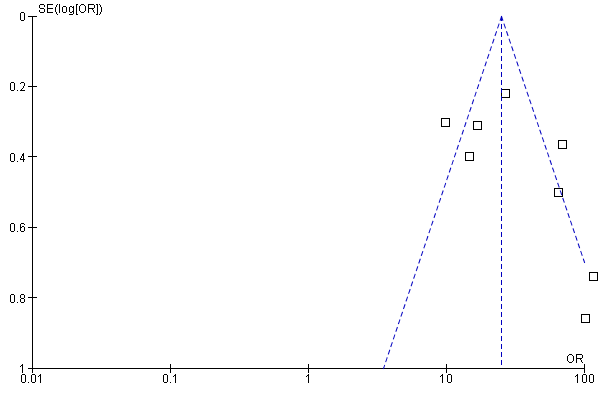


**Figure S11:** Funnel plot of the comparison between CC and CT genotypes in elite power athletes.


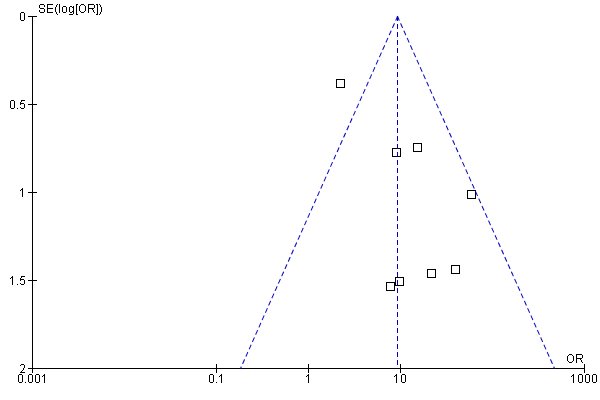


**Figure S12:** Funnel plot of the comparison between CT and TT genotypes in elite power athletes.


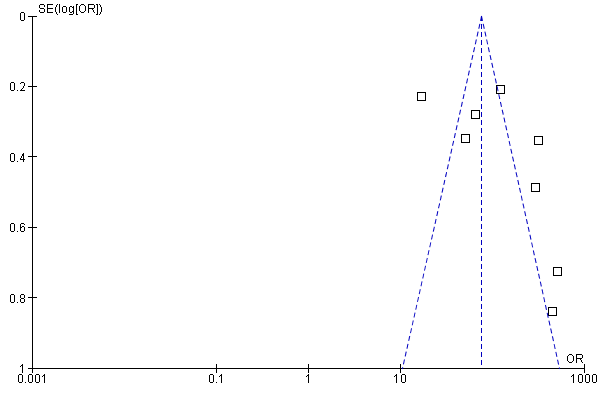


**Figure S13:** Funnel plot of the C allele versus T allele in elite power athletes.
